# Efficient Traffic Engineering in an NFV Enabled IoT System †

**DOI:** 10.3390/s20113198

**Published:** 2020-06-04

**Authors:** Thi-Thuy-Lien Nguyen, Tuan-Minh Pham

**Affiliations:** 1 University of Engineering and Technology, Vietnam National University, Hanoi 100000, Vietnam; lienntt@hnue.edu.vn; 2 Faculty of Information Technology, Hanoi National University of Education, Hanoi 100000, Vietnam; 3 Faculty of Computer Science, Phenikaa University, Hanoi 12116, Vietnam; 4 Phenikaa Institute for Advanced Study (PIAS), Phenikaa University, Hanoi 12116, Vietnam

**Keywords:** internet of things, vIoT, network functions virtualization, traffic engineering, optimization

## Abstract

The Internet of Things (IoT) is increasingly creating new market possibilities in several industries’ sectors such as smart homes, smart manufacturing, and smart cities, to link the digital and physical worlds. A key challenge in an IoT system is to ensure network performance and cost-efficiency when a plethora of data is generated and proliferated. The adoption of Network Function Virtualization (NFV) technologies within an IoT environment enables a new approach of providing services in a more agile and cost-efficient way. We address the problem of traffic engineering with multiple paths for an NFV enabled IoT system (vIoT), taking into account the fluctuation of traffic volume in various time periods. We first formulate the problem as a mixed linear integer programming model for finding the optimal solution of link-weight configuration and traffic engineering. We then develop heuristic algorithms for a vIoT system with a large number of devices. Our solution enables a controller to adjust a link weight system and update a flow table at an NFV switch for directing IoT traffic through a service function chain in a vIoT system. The evaluation results under both synthetic and real-world datasets of network traffic and topologies show that our approach to traffic engineering with multiple paths remarkably improves several performance metrics for a vIoT system.

## 1. Introduction

The Internet of Things (IoT) is an enabler of the digital revolution through which a variety of things (e.g., people, actuators, sensors) integrated over the Internet are able to interact worldwide. IoT systems have been commonly implemented in diverse fields such as the climate, education and engineering with many applications. A smart home network, for example, utilizes data gathered by a collection of home sensors and is exchanged by the cities’ information network to enhance the quality of living and energy use. As a number of connected IoT devices are growing at a breathtaking pace, the data produced, processed, or distributed by IoT devices clearly receives undivided attention. Moreover, with a large number of users in IoT networks, the usage of an independent link with each user will contribute to an unreasonable demand with Internet connectivity. Therefore, a network architecture with effective data transfer is highly required for an IoT network.

The adoption of Network Function Virtualization (NFV) technologies within an IoT environment enables a new approach of providing services in a more agile and cost-efficient way. Compared to today’s network infrastructure based on specialized appliances, Network Functions Virtualization (NFV) based on network softwarization and cloudification has emerged as a promising approach to building agile networks and reducing capital and operational expenditures. NFV enables service providers to rapidly launch and release on-demand IoT services in a flexible way. Hence, we believe that Network Functions Virtualization (NFV) is a promising technology that could help to address these challenges and leverage the IoT architecture in the Cloud era. In the system under our consideration, a network function (e.g., deep packet inspection (DPI), firewalls) or an IoT function (e.g., measurement, home automation) can be installed as software on a standardized server and linked to build an IoT service. A flow of IoT traffic is driven through a service function chain (SFC) consisting of several virtual functions (VNF) in an ordered sequence [1]. We refer to such a system as an NFV enabled IoT system (vIoT).

In this paper, we present an efficient solution to traffic engineering for improving the performance of a vIoT system. As a VNF instance is dispersed in an infrastructure, directing a flow of IoT traffic through an SFC and retaining good network performance and cost-efficiency is challenging, especially in the fluctuation of traffic volume. We aim to find a traffic engineering (TE) solution with equal-cost multipath (ECMP) for improving the performance of a vIoT system taking into account the instability of traffic demand in various periods. ECMP is a multipath routing technique that evenly splits the overall traffic passing through a network node into several minimum-cost routes. We first maximize the ratio of total satisfied traffic by considering different types of service demands, named differentiated demands. More specifically, we divide service demands into two types, where the system fully satisfies demands belonging to the first type and serves those associated with the second type with its best efforts. We then minimize the total routing cost when taking into account the dynamics of traffic demands over multiple time periods. Finally, we consider both differentiated demands and multiple time periods for optimizing multiple objectives in a TE solution.

A detailed discussion of the literature on the use of NFV for IoT has been provided in Section 2. As discussed in Section 2, much of the existing work on vIoT is concerned with the architecture design of an NFV enabled IoT system [2,3,4]. Some works have considered the performance of vIoT [5,6,7,8]. However, none of these works have addressed the optimization problem of multipath routing in a vIoT system taking into account the fluctuation of demand volume and the differentiated demands, which is important for improving the performance of a vIoT system.

The major contributions of this paper are as follows:

- We introduce the TE problem in a vIoT system and provide a mixed linear integer programming model (MILP) for finding the optimal solution of the problem. Our solution takes into consideration the ECMP routing technique, SFC, the different types of requests, and the fluctuation of traffic volume in various periods.
- In a large scenario, we suggest an effective heuristic algorithm for addressing the TE problem in a vIoT system with a large number of devices. The algorithm offers a link metric vector and a traffic splitting solution to optimize the overall traffic served and reduce the total routing cost concerning the categories of service demands, the dynamics of traffic demands, and the usable system capacity.
- We use two real network datasets and two synthetic network topologies in evaluation scenarios. We compare a number of our TE solutions, taking into consideration differentiated demands and different time periods in three major performance metrics including the routing cost, the total satisfied traffic, and the number of accepted demands. The evaluation results show that our proposal for traffic engineering of a vIoT system performs better than a scheme that ignores the classification and the fluctuation of demand volume.

The remainder of this paper is organized as follows. Section 2 provides a brief overview of related works. We describe the system and problem in Section 3. The formulation of the TE problem is presented in Section 5. We propose a heuristic algorithm for solving the large scale problem in Section 4. We evaluate our proposed model and algorithm in Section 6. Finally, conclusions are stated in Section 7.

## 2. Related Work

NFV is an attractive research area by its flexibility and agility in network designing via virtualization technology application. In recent years, researchers have studied many issues raised in NFV, including SFC routing, VNF placement, resource management, and orchestration. For example, in [9], Medhat et al. address the VNF implementation and migration issue when an instance of service functions becomes overwhelmed by seeking an appropriate way to redirect traffic flows. In [10], Eramo et al., present three algorithms that tackle VNF instance and SFC routing in reaction to changes in the workload. Some other platforms are also developed to enhance packet processing on generic servers [11,12]. In [13], Kulkarni et al. propose the NFVnice framework to improve NF performance and schedule resource efficiently, fairly and dynamically. In [14], Sun et al. also investigate to improve NFV performance by applying network function parallelism. In [15], Cotroneo et al. investigate overload control and propose a novel framework (named NFV-Throttle) to protect NFV services from failures due to an excess of traffic in the short term and to preserve the QoS of traffic flows admitted in the network. The work of [16] presents a deployment algorithm for service chains that optimizes performance by minimizing the actual cost of virtual switching. More research results about the NFV performance are presented in [17,18,19,20,21].

The scientific literature has addressed different aspects that highlight how NFV can be an enabler for future IoT platforms. For example, Ojo et al. propose an SDN-IoT architecture with NFV implementation where traditional gateways are replaced with SDN-enabled gateways [22]. Wang et al., propose the use of NFV for creating a network slice in an IoT environment and implement an IoT scenario with multiflow transmissions [23]. Mouradian et al. follow a similar objective [3]. However, they focus on the design of a distributed IoT gateway using NFV and SDN technologies for on-the-fly service provisioning in disaster management. Differently, Fu et al. design an NFV-enabled IoT framework where complex VNFs are divided into smaller VNF components, and a deep reinforcement learning scheme is used for dynamic SFC embedding [24]. In [2], Diaz et al. present some IoT challenges for the effective implementation of IoT services and highlight the use of network cloudification and softwarization that is essential to face the IoT revolution. In [25], Verma et al. investigate the resource utilization problem in a cloud environment for optimizing the allocation of virtual machines (VM) and the consolidation process. The proposed approaches can reduce remarkably the energy consumption, total VM migration, and host shutdown while maintaining the system performance. For a survey paper related to the evolution of IoT and NFV, we refer readers to the work by Farris et al. [4].

The above works focus on the architecture design of vIoT. However, they did not consider efficient algorithms to properly address, among other potential issues, service placement, scaling, and load balancing aspects, which are critical aspects affecting the performance of vIoT. In [5], Kaiwartya et al., study the optimization problem of fault tolerance for wireless sensor networks in IoT. The authors propose an adapted nondominated sorting based genetic algorithm for solving the optimization problem. However, the paper focuses on the access layer with the virtualization of sensors rather than the incorporation of NFV and IoT. Xu et al. consider the optimization problem of VNF placement for IoT applications in the mobile edge clouds. They develop both optimization models and algorithms for a special case of the problem with the bandwidth resource constraint [8]. However, they ignored the traffic fluctuations, the maximum achievable flow, and SFC constraints, which are essential for providing the performance and agility of a vIoT system. There are some research works that aim to obtain the maximum traffic flow in NFV systems. In [6], Jia et al., investigate to minimize the operational cost and maximize network throughput. Specifically, the authors consider the consumption of computing resource for the implementation of a service chain and that of bandwidth resource for data routing. They address the optimization problem of operational cost and network throughput for a sequence of requests. In [7], Sallam et al., study the SFC-constrained shortest path problem in order to find the maximum feasible flow from a source to a destination. Several studies have considered the dynamics of traffic rate in NFV networks. In [26], Ma et al., aim to minimize the overall traffic volume for a given flow when considering the variations of traffic volume after being processed by some network functions. In [27], Fei et al., consider the fluctuations of data traffic in the proactive VNF provisioning problem for NFV providers in order to minimize the cost incurred by inaccurate prediction and VNF deployment. However, the optimization model has not been provided, and multipath routing has not been considered.

To the best of our knowledge, no existing research has focused on traffic steering for improving the performance of a vIoT system taking into account multipath routing, the fluctuation of demand volume, and the differentiated demands. This paper is an extended version of our work presented at the 7th International Conference on Communications and Electronics (ICCE, 2018) [28]. Our work provides new results of optimization models and algorithms for traffic engineering in a vIoT system using multipath routing when taking into consideration the difference of service demand types and the combination of various demand types and time periods for optimizing the total satisfied traffic and reducing the network routing cost.

## 3. System Description

**Figure 1:**
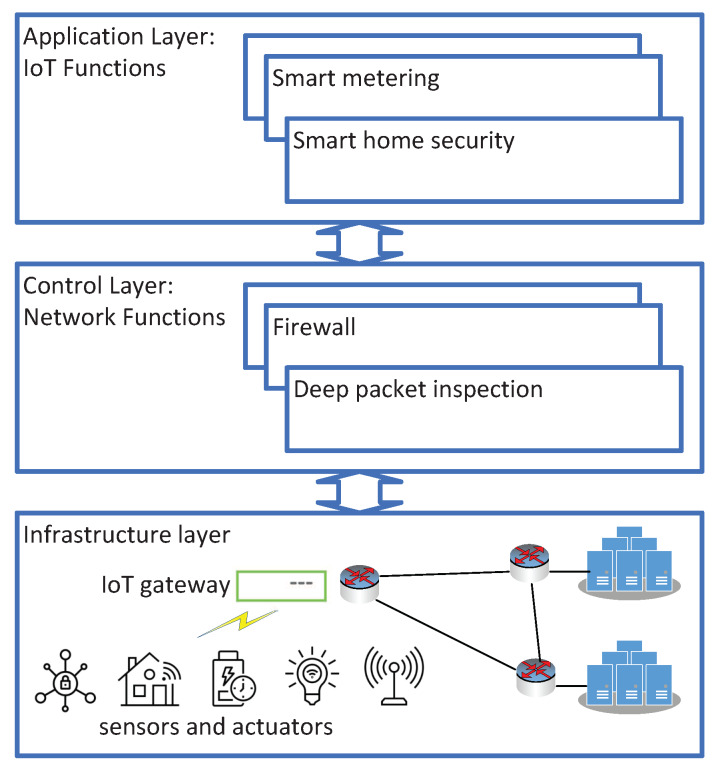
An NFV enabled IoT framework (vIoT).

We study a vIoT system that provides IoT functions and network functions deployed at the application layer and the control layer as a service (Figure 1). In vIoT, a VNF is the software implementation of network functions and IoT functions, managed by NFV Management and Orchestration (MANO) [29]. In an NFV system, an end-to-end service can be composed of several virtual functions deployed in a cloud environment (Figure 3 in [29]). As the virtualization layer of NFV is responsible for enabling the software that implements the VNF (page 18 in [29]), it can be further classified as the control layer providing network functions and the application layer providing IoT functions. A flow of IoT traffic generated by sensors at the infrastructure layer can be processed in a sequence of virtual functions such as deep packet inspection at the control layer and smart metering at the application layer. We assume that the implementation of virtual functions at NFV nodes incurs costs, and data transmission at links interconnecting the nodes also incurs communication costs.

**Figure 2:**
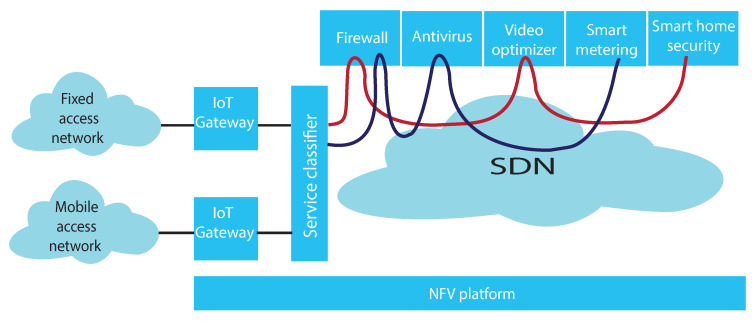
Service chain provisioning in vIoT.

A service demand in vIoT is a request from customers for a service that may include several VNFs implementing IoT and network functions. To serve a service demand, NFV MANO finds a solution of resource allocation for the implementation of a SFC requested by the service demand and routes data traffic through the SFC, as illustrated in Figure 2. We consider ECMP, a widely multipath routing strategy, for improving the performance of a vIoT system.

Our first objective is to maximize the satisfied traffic throughput in vIoT. To achieve this purpose, we consider differentiated demands in the TE problem, called Problem 1—Traffic engineering under differentiated demands (ED). Specifically, we classify a set of service demands into two types. The system fully satisfies service demands belonging to the first type and serves those associated with the second type with its best efforts. Data traffic on a link is adjusted by optimizing the link weight system of the infrastructure. Our second objective is to minimize the network routing cost in vIoT. We consider multiple time periods in the TE problem, named Problem 2—Traffic engineering under multiple time periods (EP). Particularly, we take into account the fluctuation of data traffic demand in different time periods. Finally, we consider multiple optimization objectives in the TE problem in which we take into account both differentiated demands and multiple time periods, called Problem 3—Traffic engineering under differentiated demands and multiple time periods (EDP). In the next section, we state formally the above problems and formulate them as Mixed Integer Linear Program models for finding the optimal solution.

## 4. Optimization Models

### 4.1. System Formulation

We model a vIoT system as a directed graph G=(V,E), where *V* is a set of *n* nodes and *E* is a set of *k* directed links. Let *F* be all VNFs available in the system. Node v∈V represents a commodity hardware device where one or more VNFs can be instantiated flexibly to serve demands. Node *v* has a limited capacity for instantiating VNFs and can support some functions in *F*. We use $C_{2,v}$ to denote the processing capacity of node *v*. Link e∈E is a connection between the starting node $i_{e}$ and the terminating node $j_{e}$. Let $C_{1,e}$ denote the bandwidth capacity of link *e*.

We use *D* to represent a set of *m* service demands requested by tenants. Demand *d* is defined as a customer request that requires SFC $F_{d}\subset F$ from source $s_{d}$ to destination $t_{d}$ with different bandwidth requirements $h_{d}$. We assume that link e∈E is assigned a weight and we define $w=\left\{w_{e}:e\in E\right\}$ to be the link weight system on the vIoT system. We use a traffic splitting vector $x=\left\{x_{epd}:e\in E,d\in D,p\in P_{d}\right\}$ to represent the routing solution where $P_{d}$ is all available paths on the vIoT system of demand *d* and $x_{epd}$ is the amount of data on link *e* of flow *p* of demand *d* according to the link metric vector *w*. Next, we use $r_{vf}$ to present the computing resources required for processing function *f* with one unit of traffic rate at node *v*. Others variables are as follows:

- $k_{vd_{i}}=1$ if node *v* provides the *i*th VNF in SFC $F_{d}$ of demand *d*; $k_{vd_{i}}=0$ otherwise.
- $l_{uv}$ corresponds to the smallest length of all paths from node *u* to node *v*.
- $u_{ev}=1$ if link *e* is on a minimum-cost path to node *v*; $u_{ev}=0$ otherwise. The variable is used for guaranteeing the ECMP routing strategy.
- $y_{e}$ is the total traffic through link *e*.

We provide a summary of the mathematical notations representing the vIoT system model, and the main parameters and variables of our optimal solutions in Table 1.

Now, we formally state three TE problems as follows:

- Problem 1 (Traffic engineering under differentiated demands (ED)): Given a vIoT system G=(V,E), a set of service demands *D* is classified to two types $D_{1}$ and $D_{2}$ where the system fully satisfies demands belonging to $D_{1}$, and serves those associated with $D_{2}$ with its best efforts, find a routing solution $x_{epd}$ and a link weight system $w=\left\{w_{e}:e\in E\right\}$ where e∈E, p∈P and d∈D, satisfying all requirements of the bandwidth capacity constraint on vIoT links and the computing capacity constraint on vIoT nodes in order to maximize the total satisfied traffic.
- Problem 2 (Traffic engineering under multiple time periods (EP)): Given a vIoT system G=(V,E), a set of service demands *D*, a set of path *P*, a set of periods *T* that presents for the variation in traffic demand volume and cost structure during multiple time periods, find a routing solution $x_{epdt}$ and a link weight system $w=\left\{w_{e}:e\in E\right\}$ where e∈E, p∈P, d∈D and t∈T, satisfying all requirements of the bandwidth capacity constraint on vIoT links and the computing capacity constraint on vIoT nodes in order to minimize the total network routing cost.
- Problem 3 (Traffic engineering under differentiated demands and multiple time periods (EDP)): Given a vIoT system G=(V,E), a set of service demands *D* that is classified to two types $D_{1}$ and $D_{2}$ where the system fully satisfies demands belonging to $D_{1}$, and serves those associated with $D_{2}$ with its best efforts, a set of path *P*, a set of periods *T* that presents for the variation in traffic demand volume and cost structure during multiple time periods, find a routing solution $x_{epdt}$ and a link weight system $w=\left\{w_{e}:e\in E\right\}$ where e∈E, p∈P, d∈D and t∈T, satisfying all requirements of the bandwidth capacity constraint on vIoT links and the computing capacity constraint on vIoT nodes to obtain multiple objectives: (1) to maximize the ratio of accepted demand, (2) to maximize the ratio of total satisfied traffic to total required traffic and (3) to minimize the network routing cost.

Based on NFV characteristics, resource constraints on vIoT nodes, and vIoT links, the system applies the ECMP strategy to determine x(w) for achieving the optimal routing solution with link metric vector w. In the following sections, we will present specific constraints and objective functions to obtain the optimal solution including the optimal metric of links and the best routing solution for the three problems.

### 4.2. Traffic Engineering under Differentiated Demands

In this section, we formulate the ED problem as a Mixed Integer Linear Programming (MILP) model, named ED-O, in order to obtain the optimal solution. Our aim is to find the optimal link weight system w and the best routing solution for obtaining the maximal total satisfied traffic. In this case, we consider multiple types of service demands by classifying the set of demands to two classes, $D=D_{1}\cup D_{2}$, where the system fully satisfies demands belonging to $D_{1}$ and serves those associated with $D_{2}$ with its best efforts.

The objective is to find the optimal solution for obtaining the maximal total data traffic that the network system can support. Hence, the objective function is as follows:

(1) $$F=\sum_{d,p}x_{epd}.$$

We now describe the constraints of our model. First, for constraint (2), (3) make sure that for demand $d\in D_{1}$ the total traffic outgoing from the starting node and the total traffic incoming the terminating node are the same as its traffic volume. In other words, the system supports fully for demand *d* belonging to $D_{1}$. Similarly, for constraint (4), (5) make sure that for demand $d\in D_{2}$ the total traffic outgoing from the starting node and the total traffic incoming the terminating node are no more than its traffic volume. This implies that service demand $d\in D_{2}$ allows the system to serve a portion of its traffic. The equations are as follows:

(2) $$\sum_{p,e:i_{e}=s_{d}}x_{epd}=h_{d},\;\forall d\in D_{1}$$

(3) $$\sum_{p,e:j_{e}=t_{d}}x_{epd}=h_{d},\;\forall d\in D_{1}$$

(4) $$\sum_{p,e:i_{e}=s_{d}}x_{epd}\leq h_{d},\;\forall d\in D_{2}$$

(5) $$\sum_{p,e:j_{e}=t_{d}}x_{epd}\leq h_{d},\;\forall d\in D_{2}$$

To make sure that total traffic in-flow and out-flow of each link are equal, we use constraint (6):

(6) $$\sum_{p,e:i_{e}=v}x_{epd}-\sum_{p,e:j_{e}=v}x_{epd}=0,\;\forall d,\forall v:v\neq s_{d},v\neq t_{d}$$

Next, we formulate constraint (7) to make sure that total traffic passing through a link is not greater than the bandwidth capacity of that link. The equation is given by:

(7) $$\sum_{d,p}x_{epd}\leq C_{1,e},\;\forall e$$

In addition, to represent the constraint on the computing capacity of nodes, we use constraint (9). This constraint makes sure that the VNFs deployment does not violate the computing capacity of nodes where Equation (8) computes resources required to process function *f* on node *v* per one unit of traffic. The equation is given by:

(8) $$R_{v}\left(x,f\right)=x\cdot r_{vf}$$

(9) $$\sum_{d,i}R_{v}(k_{vdi}\cdot \sum_{p,e:j_{e}=v}x_{epd},F_{di})\leq C_{2,v}$$

It is necessary to ensure that each VNF of SFC requested by demand *d* has to be implemented at one node and any flow of demand *d* has to go through its VNFs. We formulate this constraint as follows:

(10) $$\sum_{e}x_{epd}\cdot \left(k_{i_{e}d_{i}}+k_{j_{e}d_{i}}\right)>0,\;\forall d,i,t,h_{d}$$

(11) $$\sum_{e}x_{epd}>0,\;\forall d,p,h_{d}>0$$

(12) $$0\leq x_{epd}\leq M_{z}\cdot b_{epd},\;\forall d,p,e$$

Next, to make certain that the in-flow and out-flow of each node are equal, we formulate the flow constraint by the following conditions where $M_{z}$ is the largest link capacity of all links:

(13) $$x_{epd}\geq \sum_{e^{\prime }:j_{e^{\prime }}=i_{e}}x_{e^{\prime }pd}-M_{z}\left(1-b_{epd}\right),\;\forall d,e,p$$

(14) $$x_{epd}\leq \sum_{e^{\prime }:j_{e^{\prime }}=i_{e}}x_{e^{\prime }pd},\;\forall d,e,p$$

Finally, to ensure that the ECMP routing strategy is used to split traffic demand, we use constraint (15)–(17) as follows:

(15) $$0\leq g_{i_{e}v}-\sum_{p,d:t_{d}=v}x_{epd}\leq \left(1-u_{ev}\right)\sum_{d:t_{d}=v}h_{d},\;\forall v,e$$

(16) $$\sum_{p}x_{epd}\leq u_{et_{d}}\cdot h_{d},\;\forall d,e$$

(17) $$1-u_{et_{d}}\leq l_{j_{e}t_{d}}+w_{e}-l_{i_{e}t_{d}}\leq \left(1-u_{et_{d}}\right)\cdot M_{z},\;\forall d,e$$

### 4.3. Traffic Engineering under Multiple Time Periods

Because the data traffic of demands may vary during different time periods according to service requests, we consider the variations in traffic demand volume and cost structure during multiple time periods in order to achieve the minimal total network routing cost.

We now extend the ED-O formulation to find the optimal solution of the TE problem under consideration of multiple time periods, named EP-O. For more details, we use a set of $T=\left\{t_{i}i=1,2,\ldots r\right\}$ to present *r* time periods. Next, we use $C_{et}$ to denote unit routing cost of link *e* for transferring a data traffic unit at time period *t*. Because of the variation in traffic data during multiple time periods, we use a traffic volume $h_{dt}$ to present for the required data traffic of demand *d* at time period *t*.

Similarly, we introduce index *t* to constants and variables related to a time period. Specially, the flow traffic variable is $x_{epdt}$. The variables representing VNF placement are $b_{epdt}$ and $k_{vd_{i}t}$. The variable related to the minimum cost path routing is $g_{uvt}$. Hence, when we use equations and constants in this formulation, the new variables and parameters with index *t* will replace the old variables without index *t*.

Our objective is to find a solution for obtaining the minimal total routing cost. Hence, we use the following objective function:

(18) $$F=\max_{t\in T}\left(\sum_{e}y_{et}\cdot C_{et}\right)$$

where $y_{et}=\sum_{d,p}x_{epdt}$.

Note that in the EP problem we do not consider the different types of service demands. Therefore, to ensure that for time period *t* and for demand *d* the total traffic outgoing from the starting node equals to the total traffic incoming from the terminating node and both of them equal to traffic volume of demand *d*, we use the two following constraints:

(19) $$\sum_{p,e:i_{e}=s_{d}}x_{epdt}=h_{dt},\;\forall d,t$$

(20) $$\sum_{p,e:j_{e}=t_{d}}x_{epdt}=h_{dt},\;\forall d,t$$

Other constraints on flow conservation, bandwidth guarantee, and SFC are derived from the ED-O formulation. The constraints on flow conservation and bandwidth guarantee for demands in EP-O are given by (6), (7), (13), and (14) where $x_{epdt}$ and $b_{epdt}$ are used instead of $x_{epd}$ and $b_{epd}$. By replacement $k_{vd_{i}t}$ and $x_{epdt}$ for $k_{vd_{i}}$ and $x_{epd}$ respectively in constraints (9)–(12) the model ensures that every traffic flow of demand *d* is processed by all VNFs required by its SFC, and the VNFs deployment is not over the computing capacity of nodes. The conditions that guarantee the minimum cost path routing and traffic splitting are (15)–(17) where $x_{epd}$, $h_{d}$, and $g_{i_{e}v}$ are replaced by $x_{epdt}$, $h_{dt}$, and $g_{i_{e}vt}$ respectively.

### 4.4. Traffic Engineering under Differentiated Demands and Multiple Time Periods

In this case, we consider both differentiated demands and multiple time periods to obtain a link weight system and a traffic splitting solution with multiple objectives, which are as follows: maximizing the ratio of accepted demands, maximizing the total satisfied total traffic throughput, and minimizing the total network routing cost.

We extend the EP-O formulation for obtaining the optimal solution of the EDP problem, named EDP-O, by considering differentiated demands as in the ED problem. Therefore, all variables, parameters, constants and conditions are described as the EP-O formulation except conditions (19) and (20) which are replaced by the following constraints:

(21) $$\sum_{p,e:i_{e}=s_{d}}x_{epdt}=h_{dt},\;\forall d\in D_{1},\forall t$$

(22) $$\sum_{p,e:j_{e}=t_{d}}x_{epdt}=h_{dt},\;\forall d\in D_{1},\forall t$$

(23) $$\sum_{p,e:i_{e}=s_{d}}x_{epdt}\leq h_{dt},\;\forall d\in D_{2},\forall t$$

(24) $$\sum_{p,e:j_{e}=t_{d}}x_{epdt}\leq h_{dt},\;\forall d\in D_{2},\forall t$$

In addition, we use Equation (25) to calculate the ratio of accepted demands for time period *t* where $n_{at}$ is number of service demands that the system can serve for time period *t*. Equations (26) and (27) are used to estimate the ratio of total satisfied traffic and the ratio of total network routing cost for time period *t*, respectively. These equations are as follows:

(25) $$\Psi_{1t}=\frac{n_{at}}{D},$$

(26) $$\Psi_{2t}=\frac{\sum_{e,p,d}x_{epdt}}{\sum_{d}h_{dt}},$$

(27) $$\Psi_{3t}=\frac{\sum_{e,p,d}C_{et}\cdot y_{et}}{\max_{t\in T}\left(\sum_{d}h_{dt}\right)\cdot \max_{t\in T,e\in E}\left(C_{et}\right)\cdot \left(V-1\right)}$$

We use an auxiliary variable ${\bar{\Psi }}_{1}$ that is the minimum ratio of accepted demands over all time periods. The constraint is given by:

(28) $${\bar{\Psi }}_{1}\leq \Psi_{1t},\;\forall t.$$

Similarly, ${\bar{\Psi }}_{2}$ and ${\bar{\Psi }}_{3}$ are sequentially the minimum of total satisfied traffic and the maximum of total network routing cost over all time periods. We express this constraints as follows:

(29) $${\bar{\Psi }}_{2}\leq \Psi_{2t},\;\forall t,$$

(30) $${\bar{\Psi }}_{3}\geq \Psi_{3t},\;\forall t.$$

Due to the trade-off between the total traffic throughput and the total network routing cost, we use the objective function (31) to find the optimal solution where α, β, and γ are the model parameters representing the weight of the accepted demands, the total satisfied traffic, and the total network routing cost, respectively. The objective function of the problem is as follows:

(31) $$F=\alpha \cdot {\bar{\Psi }}_{1}+\beta \cdot {\bar{\Psi }}_{2}-\gamma \cdot {\bar{\Psi }}_{3}$$

Our MILP formulations of three problems allow us to achieve optimal solutions and can be effectively solved for moderate networks by a MILP solver (e.g., CPLEX). Unfortunately, finding optimal link weight system of the shortest-path routing problem for an arbitrary network is NP hard [30]. Hence, we propose a heuristic algorithm to solve the TE problems for a large-scale vIoT system in the sequel.

## 5. Heuristic Algorithm

We propose a heuristic algorithm for finding an approximate solution of the three problems in a large-scale vIoT system. The objectives are as follows: (1) maximizing the total satisfied traffic, (2) maximizing the number of accepted service demands, and (3) minimizing the total network routing cost in the vIoT system. The proposed algorithm contains two primary components: (i) optimizing the link weight system while considering the differentiated demands, the dynamics of service demands, and available resources (i.e., Algorithm 1: Optimizing the link weight system); (ii) deciding a routing solution based on the link weight system vector, service demands, and available system resources by the ECMP technique (i.e., Algorithm 2: TrafficDecision, Algorithm 3: VNFAllocation). The algorithm is deployed as a component in the NFV orchestrator block of the NFV reference architecture framework (Figure 4 in [29]).

**Figure 3:**
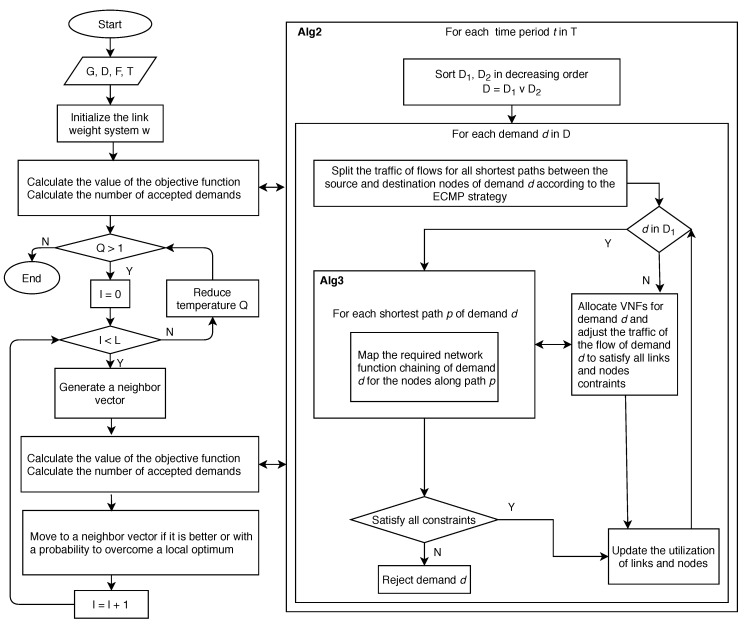
Flowchart of the proposed algorithm.

The main steps of the proposed algorithm are presented as a flow chart in Figure 3. In the sequel, we present the details of Algorithms 1–3.
**Algorithm 1** Optimizing the link weight system**Input:***G, D, F, T* **Output:** Routing solution x, link weight system w
1:Initialize w2:$\left(O_{w},A_{w}\right)\leftarrow$ TRAFFICDECISION(G,D,F,T,w)3:$w_{best}\leftarrow w$, $O_{best}\leftarrow O_{w}$, $A_{best}\leftarrow A_{w}$4:$Q\leftarrow Q_{o}$5:**while** Q>1 **do**
6:    l←07:    **while** l<L **do**8:        z←generateaneighborvector9:        $\left(O_{z},A_{z}\right)\leftarrow$ TRAFFICDECISION(G,D,F,T,z)10:        $\Delta O\leftarrow O_{z}$ - $O_{w}$, $\Delta A\leftarrow A_{z}$ - $A_{w}$11:        **if** ΔO≤0 **and** ΔA≥0 **then**12:            w←z, $O_{w}\leftarrow O_{z}$, $A_{w}\leftarrow A_{z}$13:            **if** $O_{z}<O_{best}$ **then**14:                $w_{best}\leftarrow z$, $O_{best}\leftarrow O_{z}$, $A_{best}\leftarrow A_{z}$15:            **end if**16:        **else**17:            **if** $e^{-\Delta O/Q}>$ random(0,1) **then**18:                w←z, $O_{w}\leftarrow O_{z}$, $A_{w}\leftarrow A_{z}$19:            **end if**20:        **end if**21:        l←l+122:    **end while**23:    reduce temperature *Q*24:**end while**


### 5.1. Optimizing the Link Weight System

In this component, we propose an approximation algorithm based on Simulated Annealing heuristic [31] to find the optimal link weight system (Algorithm 1). First, the algorithm generates an initial link metric vector where all link weights equal to 1. The algorithm then optimizes the metric of links by two loops underlying the neighborhood selection scheme. Specially, for two loops, we use *Q* and *L* as two parameters to set up the initial temperature and the times of the inner while-end loop, respectively. For generating a new neighborhood (i.e., line 8), the algorithm increases the weight of links whose routing cost is highest. Next, based on the new neighborhood vector *z*, the algorithm uses the TrafficDecision function to find a routing solution and to estimate the link metric vector *z* based on the objective function $O_{z}$. The detail of TrafficDecision is presented in Algorithm 2 and is described in the following section. The new link weight system *z* is better than the current link weight vector if and only if the value of the objective function $O_{z}$ is not worse and the achieved minimum accepted demands do not decrease. The optimization process updates the current vector in the next loop by the neighborhood vector *z* if it is better or with probability $e^{-\Delta O/Q}$ to overcome a local optimum. Finally, the algorithm achieves the optimal link weight system to update the vIoT system.
**Algorithm 2** Deciding a traffic splitting vector1:**function**TrafficDecision(G,D,F,T,w)2:    **for all** t∈T **do**3:         sort $D_{1}$ decreasingly by traffic volumes4:         sort $D_{2}$ decreasingly by traffic volumes5:        $D=D_{1}\cup D_{2}$6:        **for all** d∈D **do**7:           $P_{d}\leftarrow$ all shortest paths for demand *d*8:           x ← split flow traffic for $P_{d}$ with ECMP strategy9:           **if** $d\in D_{1}$ **then**10:                VNFALLOCATION $\left(G,x,d,P_{d}\right)$11:               **if** satisfy all link and node constraints **then**12:                   update the utilization of links and nodes13:               **else**14:                   reject demand *d*15:               **end if**16:           **else**17:               **do**18:                    VNFALLOCATION $\left(G,x,d,P_{d}\right)$19:                   **if** do not satisfy all link and node constraints **then**20:                       decrease flow traffic x21:                   **else**22:                       increase flow traffic x23:                   **end if**24:               **while** do not satisfy all constraints25:               update the utilization of links and nodes26:           **end if**27:        **end for**28:        ${\bar{A}}_{t}\leftarrow$ compute number of accepted demands at time period *t*29:    **end for**30:    $A_{w}\leftarrow$$\min_{t\in T}{\bar{A}}_{t}$31:    $O_{w}\leftarrow$ compute value of the objective function    **return** $O_{w},A_{w}$
32:**end function**

### 5.2. Deciding the Traffic Splitting Vector

With a metric of links on the vIoT system, we propose the TrafficDecision algorithm to determine the traffic splitting vector based on the ECMP technique, SFC, and resource constraints. The detail of TrafficDecision is presented in Algorithm 2. Specially, the first step in the algorithm is to find all shortest paths from the starting node to the terminating node of demands. Next, TrafficDecision applies the ECMP technique to determine how to split the traffic volumes of demands on these paths. Then, the algorithm finds an allocating VNFs solution for nodes along these paths using VnfAllocation function, presented in Algorithm 3. The VnfAllocation function considers mapping the required network function chaining of each demand for the nodes along each path sequentially. When it cannot find a possible traffic splitting solution for a demand, this demand is rejected if it belongs to class $D_{1}$ or its traffic is adjusted if it belongs to class $D_{2}$. All of these steps are looped through all demands for each time period.

As mentioned above, this component is modified when applying to find an approximate solution for ED, EP, and EDP. Specifically, we use Algorithm 2 to find the traffic splitting vector for the EDP problem. For the ED problem, we modify Algorithm 2 by removing some lines, including line 2 and 29. Similarly, we apply Algorithm 2 after removing some lines from line 16 to 26 and line 9 for solving the EP problem.
**Algorithm 3** Allocating VNFs of demands1:**function**VnfAllocation($G,x,d,P_{d}$)2:    **for all** $p\in P_{d}$ **do**3:        $\bar{V}\leftarrow$ all nodes belongs to *p*4:        $\bar{F_{d}}\leftarrow$ all network functions of $F_{d}$5:        **while** $\bar{V}$ is not empty and $\bar{F_{d}}$ is not empty **do**6:           v← the first node of $\bar{V}$7:           remove the first node from $\bar{V}$8:           **while** available capacity of v>0 and $\bar{F_{d}}$ is not empty **do**9:               f← the first function of SFC $\bar{F_{d}}$10:               **if** available capacity of $v\geq R_{v}\left(x_{epd},f\right)\;\mathrm{then}$11:                   mapping *f* for *v*12:                   remove the first function from SFC $\bar{F_{d}}$13:               **else**14:                   break15:               **end if**16:           **end while**17:        **end while**18:    **end for****return***G*19:**end function**

## 6. Evaluation

In this section, we present the scenarios and parameters in our experiments using both real-world datasets and synthetic topologies to evaluate the performance of our solution with multiple objectives. First, we evaluate the efficiency of our TE solution when considering differentiated demands. Second, the simulations are focused on comparing the multiple time periods scheme with the nonmultiperiod scheme. Finally, we evaluate the strategy considering both differentiated demands and multiple time periods.

### 6.1. Scenario Setting

To evaluate performance of our solution, we use four scenarios as given in Table 2, where the Internet2 dataset [32] and the GEANT dataset [33] are real-world datasets and the later ones are random generated by synthetic topologies based on the two-tier model [34] and the Bcube model [35], sequentially.

For more details, the Internet2 dataset includes a topology that consists of 12 nodes, 30 links, and 120 service demands. In this dataset, we consider three time periods where the traffic volumes of demands in the first time period are recorded from the real traffic, and the others are generated from the minimum to the maximum traffic volume of all demands randomly. The Geant dataset contains a topology that consists of 22 nodes, 72 links, 200 service demands, and four time periods. Similarly, for the first time period we use the traffic volumes of demands that tracked from the Geant network, and the traffic demands in other time periods are randomly generated. The third and fourth datasets are randomly generated by FNSS tool [36] based on the Two-tier and Bcube models, respectively. The Two-tier dataset contains a topology of 60 nodes and 282 links and traffic matrices with 40 service demands. The Bcube dataset is a network consisting of 176 nodes and 1464 links with 200 service demands. In all four experiments, we consider four VNFs available on the vIoT system.

The processing capacity of a node, the resource requirements of a VNF, and the SFC of a demand are randomly generated with the Uniform distribution. A demand will be classified to $D_{1}$ and $D_{2}$ based on the average of its traffic volumes over all time periods. If the average is larger than the average of all traffic volumes of all demands over all time periods, a demand will belong to $D_{2}$. We set α = 3, β =2, γ = 1 since we consider that the number of accepted demand has the highest priority and the total routing cost has the lowest priority.

In the evaluation, we consider three performance metrics: the ratio of accepted demands, the ratio of total satisfied traffic, and the total network routing cost. Specifically, the ratio of accepted demands is the proportion of a number of service demands supported by the system to that requested by customers. Similarly, the ratio of total satisfied traffic is the proportion of total traffic provided by the system to that required by customers. The total network routing cost is the sum of routing cost of all links used in the system where the routing cost of link *e* is the product of the total traffic through link *e* and the unit routing cost of link *e*.

### 6.2. Performance Evaluation of the Traffic Engineering Solution Considering Differentiated Demands

We evaluate the efficiency of the proposed algorithms for the ED problem, whose objective is to maximize the total satisfied traffic that the system supports all service demands. For simplicity, we refer to this algorithm for schemes considering classified demand types and nonclassified demand types as multitype and one-type sequentially. We perform our solution in the same set of demands and network scenarios for two cases. In the multitype case, we use two demand types where the system fully satisfies demands belonging to the first type, and serves those associated with the second type with its best efforts.

**Figure 4:**
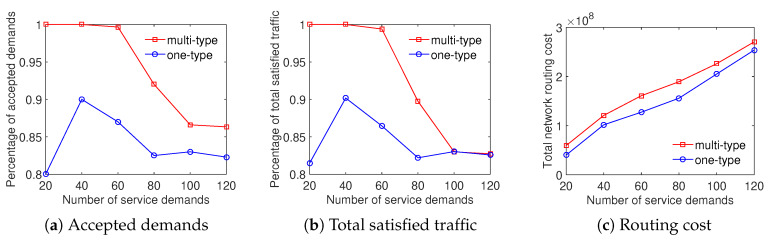
Efficient traffic engineering solution considering differentiated demands with the Internet2 dataset.

**Figure 5:**
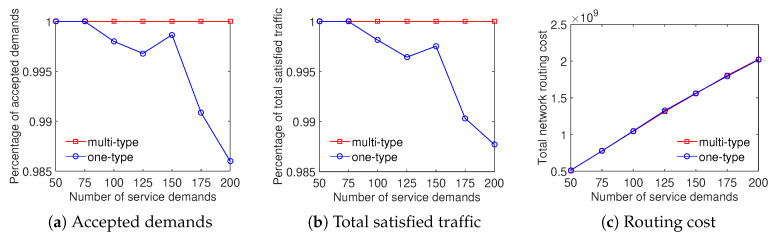
Efficient traffic engineering solution considering differentiated demands with the Bcube dataset.

**Figure 6:**
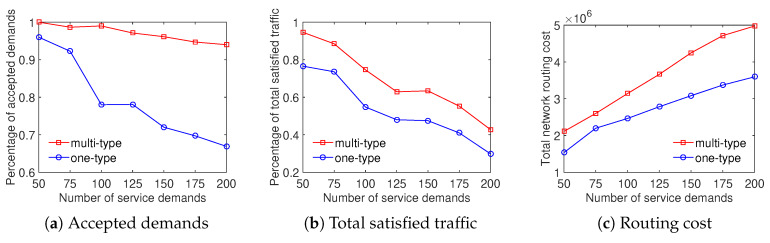
Efficient traffic engineering solution considering differentiated demands with the GEANT dataset.

**Figure 7:**
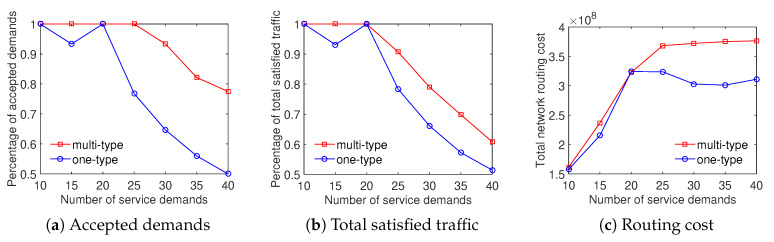
Efficient traffic engineering solution considering differentiated demands with the Two-tier dataset.

In four scenarios, the experiment results show that the Multi-type scheme outperforms the One-type scheme in both the total satisfied traffic objective and the ratio of accepted demands objective. Particularly, Figure 4 shows that the ratio of satisfied demands achieved by the Multi-type scheme is higher from 5% to 25% than that of the One-type scheme for the Internet2 dataset. The result of the percentage of total satisfied traffic is similar in this case. It can be seen from Figure 4c that the Multi-type scheme achieves a higher total network routing cost than the One-type scheme. The main reason is that the Multi-type scheme supports for much more network throughput therefore its resource utilization is larger. We can observe that the Multi-type scheme overcomes the One-type scheme not only the accepted demands and total traffic but also the total network routing cost in Figure 5 with the Bcube dataset. Moreover, the gap between the effective performance of the Multi-type scheme and the One-type scheme becomes larger with the increase of number of service demands when evaluating the ratio of supported demands and total satisfied traffic in Figure 5a,b. Figure 6 and Figure 7 illustrate the similar results for the Geant and Two-tier datasets. To summarize, the approach considering differentiated demands is effective in maximizing the total satisfied traffic.

### 6.3. Performance Evaluation of the Traffic Engineering Solution Considering Multiple Time Periods

We evaluate the performance of our approach by comparing results achieved by the case of multiperiod and the none-period case. Note that in the multiperiod case, we divide time to multiple time periods where the traffic demand and routing cost of links may vary during different time periods. Hence, there are different values of the ratio of accepted demand, the ratio of total satisfied traffic, and the total network routing cost for different time periods. Then we use the worst one in all time periods for each aspect in the multiperiod case. In the none-period case, each service demand has only one traffic volume that is the maximal demand volume over all time periods in the multiperiod case. It is similar for the routing cost of network links. We compare three performance metrics, including the minimum ratio of accepted demand, the minimum ratio of total satisfied traffic, and the maximum total network routing cost over all time periods of the multiperiod case to that of the none-period case. The main objective of the EP problem is to minimize the total network routing cost.

**Figure 8:**
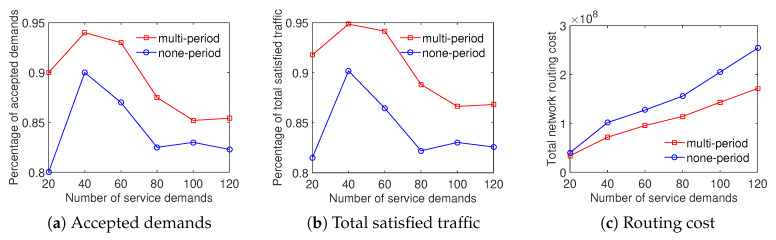
Efficient traffic engineering solution considering multiple time periods with the Internet2 dataset.

**Figure 9:**
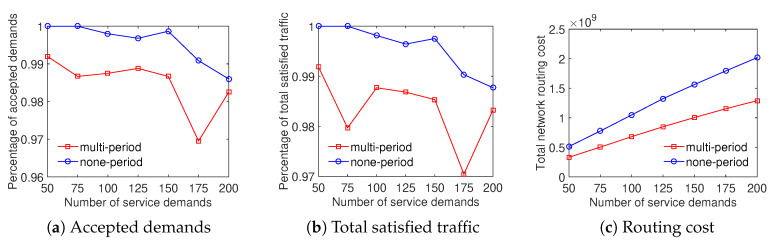
Efficient traffic engineering solution considering multiple time periods with the Bcube dataset.

**Figure 10:**
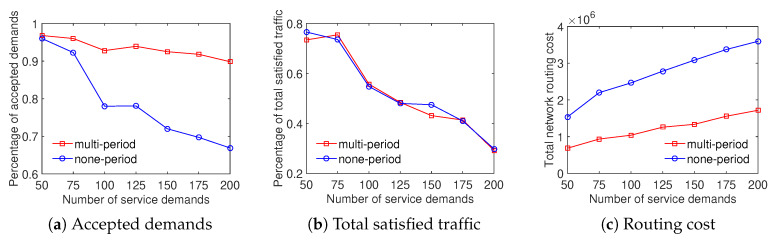
Efficient traffic engineering solution considering multiple time periods with the GEANT dataset.

**Figure 11:**
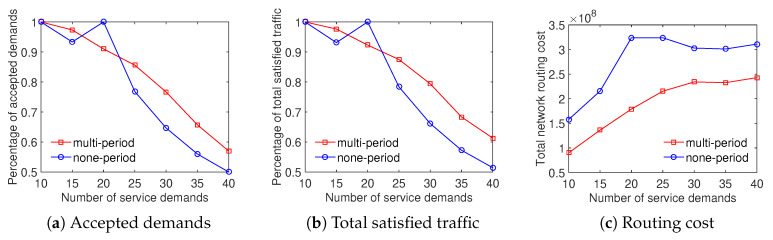
Efficient traffic engineering solution considering multiple time periods with the Two-tier dataset.

The evaluation results show that the multiperiod scheme outperforms the none-period scheme in terms of three metrics. Specially, Figure 8 depicts that the multiperiod strategy can save approximately 25% the total network routing cost and around 7% the minimum ratio of accepted demands with the Internet2 dataset. In addition, the ratio of total satisfied traffic of the multiperiod scheme is 5–13% higher than that of the none-period scheme. For the evaluation using datasets of Bcube, Geant, and two-tier, Figure 9, Figure 10 and Figure 11 depict that the multiperiod scheme saves approximately 50% of the total network routing cost compared with the none-period scheme. Similarly, the multiperiod scheme obtains a higher percentage of accepted demands and total satisfied traffic in comparison with the nonperiod scheme. To sum up, the traffic engineering solution considering multiple time periods is an effective approach to minimizing the total network routing cost.

### 6.4. Performance Evaluation of the Traffic Engineering Solution Considering the Combination of Differentiated Demands and Multiple Time Periods

We then evaluate the performance of the strategy considering both differentiated demands and multiple time periods (called COM) against the scheme considering multiple time periods only (called MP). For more details, in the first case, we aggregate both the differentiated demands scheme and the multiple time periods scheme to the algorithm. For the second case, we only consider multiple time periods scheme. However, notice that we use the new objective function as Equation (31) for the MP case that leads to the differences of results compared with the mentioned multiple time periods results.

**Figure 12:**
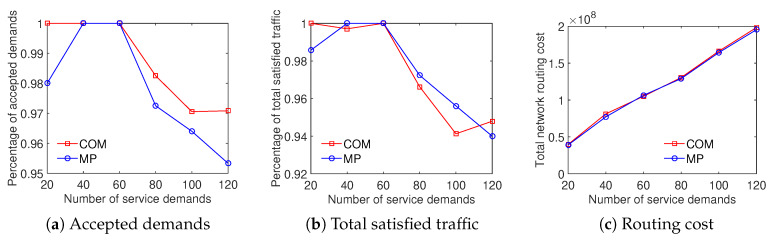
Efficient traffic engineering solution considering the combination of differentiated demands and multiple time periods with the Internet2 dataset.

**Figure 13:**
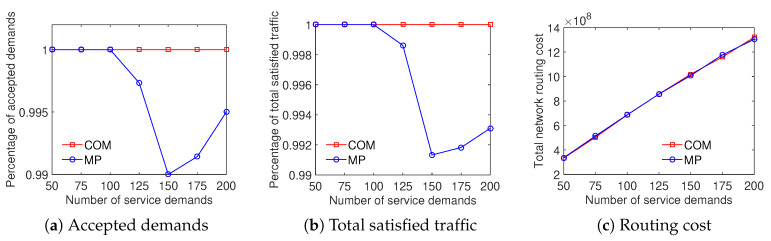
Efficient traffic engineering solution considering the combination of differentiated demands and multiple time periods with the Bcube dataset.

**Figure 14:**
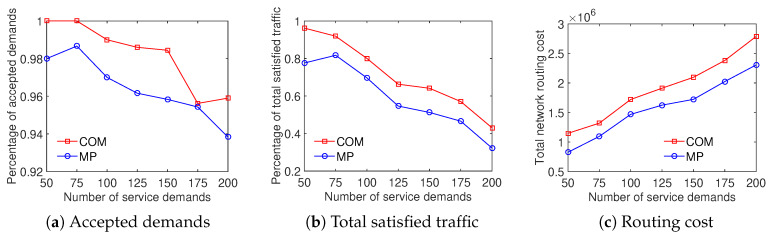
Efficient traffic engineering solution considering the combination of differentiated demands and multiple time periods with the GEANT dataset.

**Figure 15:**
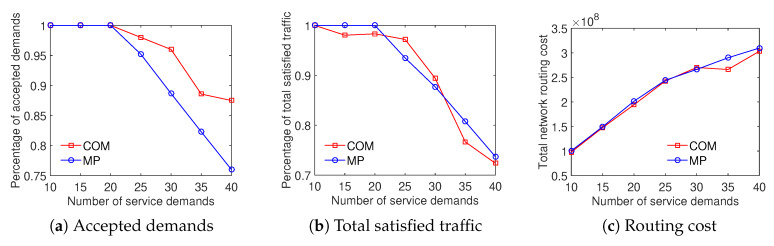
Efficient traffic engineering solution considering the combination of differentiated demands and multiple time periods with the Two-tier dataset.

The results illustrate that the COM scheme achieves a higher ratio of accepted demands than the MP scheme while both the total satisfied traffic and the total network routing cost results are equivalent. As shown in Figure 12, the ratio of accepted demands obtained by the COM scheme is better than that of the MP scheme with the Internet2 dataset. In addition, on the percentage of total satisfied traffic and the total network routing cost aspects, the results are very closed. Figure 13 compares the traffic solution considering the COM scheme to the MP scheme with the Bcube dataset. It shows that COM outperforms MP in both the ratio of accepted demands and total satisfied traffic while having the same total network routing cost. For the Geant dataset, Figure 14 shows that the COM scheme achieves at least 20% higher ratio of total satisfied traffic than the MP scheme with a number of service demands from 50 to 200. The total network routing cost of COM is higher than that of MP because of the extreme increment of satisfied traffic. The comparison between COM and MP with the two-tier dataset also shows a similar result, presented in Figure 15. To summarize, the aggregation of the multiple time periods scheme and the differentiated demands scheme provides an effective solution for traffic engineering with multiple objectives.

## 7. Conclusions

In this paper, we investigated the TE problem considering the ECMP routing technique, SFC, differentiated demands in various periods, and the fluctuation of traffic volume in a vIoT system. We proposed three MILP models for achieving the optimal link weight configuration and routing solution for the TE problem. We then developed efficient algorithms that provide an approximate solution for the large scale problem. We evaluated the performance of the proposed algorithms through simulations using both synthetic and real-world datasets of network traffic and topologies. The evaluation results demonstrate that a traffic engineering strategy aggregating both the fluctuation of demand volume and the different demand types can significantly increase the performance of a vIoT system thanks to the offered features of service composition agilely created in NFV. There are several interesting extensions of our TE approaches, including consideration of failure scenarios, traffic prediction, and cache optimization for further improving the performance of an IoT system based on NFV [17,37,38]. Another important direction might be the performance evaluation of energy consumption in a vIoT system taking into account the mobility of IoT devices.

## Figures and Tables

**Table 1:** Summary of notations.

**Sets**	
*D*	Service demands
$D_{1}$	Service demands require that the system fully satisfies their traffic volumes
$D_{2}$	Service demands allow a system to serve with its best efforts
*E*	Links on the vIoT system
*V*	Nodes on the vIoT system
*F*	VNFs available on the vIoT system
*P*	Possible paths on the vIoT system
$P_{d}$	Possible paths for demand *d*
*T*	Time periods
**Parameters**	
$h_{dt}$	The traffic volume of d∈D at time period t∈T
$s_{d}$	The starting node of demand d∈D
$t_{d}$	The terminating node of demand d∈D
$F_{d}$	The SFC requested by demand *d*, $F_{d}\subset F$
$k_{vd_{i}t}$	Node *v* supports the *i*th VNF in SFC $F_{d}$ of demand *d* at time period *t* if $k_{vd_{i}t}=1$, otherwise $k_{vd_{i}t}=0$
$C_{1,e}$	Bandwidth capacity of link e∈E
$C_{et}$	Network routing cost of link e∈E at time period t∈T
$C_{2,v}$	Computing capacity of node v∈V
$M_{z}$	The largest link capacity of all links
α	The weight of accepted demands
β	The weight of total satisfied traffic
γ	The weight of total network routing cost
**Binary variables**	
$b_{epdt}$	A binary variable represents that link *e* is on flow *p* of demand *d* at time period *t* if $b_{epdt}=1$, otherwise $b_{epdt}=0$
$u_{ev}$	A binary variable represents that link *e* is on a minimum-cost path to node *v* if $u_{ev}=1$, otherwise $u_{ev}=0$
**Continuous variables**	
$x_{epdt}$	The traffic rate on link *e* of data flow *p* of demand *d* at time period t∈T
$l_{uv}$	The smallest length of the paths from node *u* to node *v*
$y_{et}$	Total of traffic through link *e* at time period t∈T
$g_{uvt}$	Total traffic volume assigned to outgoing links of node *v* that belongs to the minimum-cost paths from node *u* to node *v* at time period t∈T
$\Psi_{1t}$	Percentage of accepted demands at time period t∈T
$\Psi_{2t}$	Percentage of total satisfied traffic at time period t∈T
$\Psi_{3t}$	Percentage of total network routing cost at time period t∈T
**Discrete variables**	
$n_{at}$	Number of accepted demands at time period t∈T
**Auxiliary variables**	
${\bar{\Psi }}_{1}$	The minimum ratio of accepted demands over all time periods
${\bar{\Psi }}_{2}$	The minimum of total satisfied traffic over all time periods
${\bar{\Psi }}_{3}$	The maximum of total network routing cost over all time periods
**Metric vector variables**	
w	The metric of links on the vIoT system, $w=\left(w_{e}:e\in E\right)$
x	A traffic splitting vector $x=\left(x_{epdt}:e\in E,p\in P,d\in D,t\in T\right)$

**Table 2:** Scenarios.

Scenarios	Number of Virtual Nodes	Number of Virtual Links	Number of VNFs	Number of Time Periods	Min Number of Demand	Max Number of Demand
Internet2	12	30	4	3	20	120
Geant	22	72	4	4	50	200
Two-tier	60	282	4	3	10	40
Bcube	176	1464	4	3	50	200
